# A simple and reproducible scoring system for EGFR in colorectal cancer: application to prognosis and prediction of response to preoperative brachytherapy

**DOI:** 10.1038/sj.bjc.6603619

**Published:** 2007-02-20

**Authors:** I Zlobec, T Vuong, S Hayashi, D Haegert, L Tornillo, L Terracciano, A Lugli, J Jass

**Affiliations:** 1 Department of Pathology, McGill University, Montreal, Québec, Canada; 2 Department of Radiation Oncology, McGill University Health Centre, Montreal, Québec, Canada; 3 Institute of Pathology, University Hospital of Basel, Basel, Switzerland

**Keywords:** EGFR, colorectal cancer, ROC curve analysis, tissue microarray, scoring system

## Abstract

The aim of this study was to determine the predictive and prognostic value of epidermal growth factor receptor (EGFR) expression in rectal cancers treated with preoperative high-dose rate brachytherapy and in mismatch-repair (MMR)-proficient colorectal cancers (CRCs), respectively. We validate the use of receiver operating characteristic (ROC) curve analysis to select cutoff scores for EGFR overexpression for the end points studied. Immunohistochemistry (IHC) for EGFR was performed on 82 rectal tumour biopsies and 1197 MMR-proficient CRCs using a tissue microarray. Immunoreactivity was scored as the percentage of positive tumour cells by three pathologists and the inter-observer reliability was assessed. ROC curve-derived cutoffs were used to analyse the association of EGFR overexpression, tumour response and several clinicopathological features including survival. The scoring method was found to be reproducible in rectal cancer biopsies and CRCs. The selected cutoff scores from ROC curve analysis for each clinicopathological feature were highly consistent among pathologists. EGFR overexpression was associated with response to radiotherapy (*P*-value <0.001) and with worse survival time (*P*-value <0.001). In multivariate analysis, EGFR overexpression was independently associated with adverse prognosis (*P*-value <0.001). Epidermal growth factor receptor is a predictive marker of response to preoperative radiotherapy and an independent adverse prognostic factor CRC.

Epidermal growth factor receptor (EGFR) is a 170-kDa transmembrane glycoprotein/cell surface receptor composed of an extracellular ligand-binding domain, a transmembrane lipophilic segment and an intracellular tyrosine kinase (Grant *et al*, 2002). Epidermal growth factor receptor belongs to the ErbB tyrosine kinase receptor family, which includes four proteins encoded by the c-erb B proto-oncogene, namely ErbB1 (EGFR), ErbB2 (HER2/neu), ErbB3 (HER3) and ErbB4 (HER4) (Yarden and Sliwkowski, 2001; Baselga, 2002). Ligand binding produces dimerisation of the receptor and activation of intrinsic protein tyrosine kinase activity leading to the transduction of signalling pathways involved in proliferation, cell division and differentiation (Herbst, 2004). The MAP kinase and AKT signalling pathways have been found to mediate intracellular EGFR signalling (Herbst, 2004). The biologic responses to MAP kinase induction result in increased expression of proteins governing cell-cycle regulation. AKT, an anti-apoptotic kinase, is implicated in cell survival and promotion of angiogenesis and has also been linked to activation of matrix metalloproteinase protein facilitating tumour growth and promotion (Kainulainen *et al*, 2000; Thant *et al*, 2000).

Expression of EGFR is linked to poor survival in a variety of malignancies (Neal *et al*, 1990; Tateishi *et al*, 1990; Nicholson *et al*, 1991; Chua *et al*, 1996; Jonjic *et al*, 1997; Gamboa-Dominguez *et al*, 2004). In colorectal cancer (CRC), it is well documented that EGFR expression may be associated with an advanced disease stage (Gross *et al*, 1991; Radinsky, 1995; Radinsky *et al*, 1995; Prewett *et al*, 2002). However, these results remain controversial because an association between EGFR expression and Dukes stage or length of survival in CRC has not been detected in other studies (Yasui *et al*, 1988; Moorghen *et al*, 1990; Koenders *et al*, 1992; Saeki *et al*, 1995; McKay *et al*, 2002).

Among the standard techniques such as protein expression, RNA transcript and DNA assays used to detect EGFR expression in tumours, immunohistochemistry (IHC) is the most commonly used in CRC (Italiano, 2006). EGFR expression had been reported in 25–82% of CRCs (Wan *et al*, 1988; Radinsky *et al*, 1995; Goldstein and Armin, 2001; Yarden and Sliwkowski, 2001; McKay *et al*, 2002; Cunningham *et al*, 2004; Spano *et al*, 2005a).

It has been recognised that the wide range of methods for interpreting EGFR expression as determined by IHC considerably hinders a meta-analysis of the predictive or prognostic value of the protein in CRC (Italiano, 2006). Despite its subjective nature, staining intensity has become an integral component of many EGFR scoring systems (Goldstein and Armin, 2001; Resnick *et al*, 2004; Italiano *et al*, 2005; Spano *et al*, 2005b). It has recently been shown, however, that the degree of staining intensity may be affected by varying fixation methods and laboratory procedures and is reduced dramatically with increased storage time of the tissue samples (Atkins *et al*, 2004; Italiano *et al*, 2006). Scoring methods for EGFR include those evaluating only the degree of staining intensity (Resnick *et al*, 2004), those for which positive or negative expression of EGFR are based on a predetermined and often arbitrarily set cutoff score (Goldstein and Armin, 2001; Umemura *et al*, 2004; Azria *et al*, 2005; Italiano *et al*, 2005; Bibeau *et al*, 2006) and those with composite systems incorporating both the extent of positivity and staining intensity (Spano *et al*, 2005b). Rarely is the choice of scoring method, in particular the selection of cutoff scores for positivity, addressed and many remain unvalidated.

The aim of this study was to determine the predictive value of EGFR in rectal cancer treated with a novel preoperative radiotherapy protocol, namely high-dose rate endorectal brachytherapy (HDREB) and its prognostic value in 1197 mismatch-repair (MMR)-proficient CRCs using the tissue microarray (TMA) technique. In pursuing this aim, we propose and validate the application of receiver operating characteristic (ROC) curve analysis to the selection of cutoff scores for EGFR overexpression for the end points under investigation.

## MATERIALS AND METHODS

### Study Group 1

This study was approved by the Institutional Review Board and informed written consent was obtained from 82 patients with rectal adenocarcinoma undergoing preoperative HDREB (Vuong *et al*, 2002; Vuong *et al*, 2005). Clinical staging according to the International Union against Cancer Classification was carried out by both endorectal ultrasonography and MRI. In cases of discrepancy, the higher T stage was assigned. Patients with abdominal nodal disease were excluded from the study, as were patients with distant metastases. Radiation was delivered preoperatively with an eight-channel endorectal catheter using a high-dose rate remote after-loading system. A daily fraction of 6.5 Gy was administered over 4 consecutive days to a total of 26 Gy. Treatment was planned using a CT simulator to obtain optimal conformal dosimetry. The dose was prescribed to a clinical target volume that included the gross tumour volume and any intra-mesorectal deposits visible at MRI. Patients underwent cancer-directed surgery 4–8 weeks following radiotherapy regardless of tumour response.

Tumours were considered completely responsive to preoperative HDREB when no histologic evidence of residual carcinoma could be pathologically determined from postoperative surgical resections (ypT0). Partial response was characterised by the presence of microfoci or foci of residual carcinoma measuring 0.3–0.9 cm in diameter, whereas no response was defined by large areas of residual carcinoma that could be identified macroscopically and ranged in size from 2 to 6 cm following irradiation.

### Study Group 2

A TMA of 1420 unselected, nonconsecutive CRCs was constructed (Sauter *et al*, 2003). Briefly, formalin-fixed, paraffin-embedded tissue blocks of CRC resections were obtained. One tissue cylinder with a diameter of 0.6 mm was punched from morphologically representative tissue areas of each donor tissue block and brought into one recipient paraffin block (3 × 2.5 cm) using a homemade semiautomated tissue arrayer.

The clinicopathological data for 1420 patients included T stage (T1, T2, T3 and T4), N stage (N0, N1 and N2), tumour grade (G1, G2 and G3), vascular invasion (presence or absence) and 10-year survival. The distribution of these features has been described previously (Lugli *et al*, 2006b).

### IHC

The 1420 CRCs were dewaxed and rehydrated in dH_2_O. Endogenous peroxidase activity was blocked using 0.5% H_2_O_2_. The sections were incubated with 10% normal goat serum (Dako Cytomation, Carpinteria, CA, USA) for 20 min. To determine MMR status, the 1420 CRCs were incubated with primary antibody for MLH1 (MLH1 clone MLH-1, BD Biosciences Pharmingen, San Jose, CA, USA), MSH2 (clone MSH-2, BD Biosciences Pharmingen, San Jose, CA, USA), and MSH6 (clone 44, Transduction Laboratories, San Diego, CA, USA) for 2 h at room temperature. Subsequently, sections were incubated with HRP-conjugated secondary antibody (K4005, EnVision+ System-HRP (AEC); DakoCytomation, Carpinteria, CA, USA) for 30 min at room temperature. For visualisation of the antigen, the sections were immersed in 3-amino-9-ethylcarbazole+substrate-chromogen (DakoCytomation) for 30 min, and counterstained with Gill's haematoxylin.

IHC for EGFR (clone 3C6, 3 mg ml^−1^, Ventana Medical Systems, Tucson, USA) was performed on the 82 pretreatment rectal tumour biopsies as well as on all 1420 CRCs using an autostainer according to the manufacturer's recommendations. Positive controls consisted of normal oral mucosa. Negative controls were treated identically with the primary antibody omitted.

### Evaluation of IHC

EGFR immunoreactivity was evaluated as either membranous or cytoplasmic in a semiquantitative manner using the proportion of EGFR-positive tumour cells over the total number of tumour cells ranging from 0 to 100%. Scores were based on 5% intervals (0, 5, 10%, etc). The rectal tumour biopsies were evaluated by three experienced pathologists (AL, JJ, SH) as were the TMA CRCs (AL, JJ, DH). For the 1420 CRCs, MLH1, MSH2 and MSH6 were scored as negative (0% staining) or positive (>0% staining). Staining intensity was not evaluated.

### MMR status

The 1420 CRCs were stratified according to DNA MMR status and consisted of 1197 MMR-proficient tumours expressing MLH1, MSH2 and MSH6, 141 MLH1-negative tumours and 82 presumed HNPCC cases demonstrating loss of MSH2 and/or MSH6 at any age, or loss of MLH1 at <55 years (Hampel *et al*, 2005). Only MMR-proficient tumours were included in this study to ensure a uniform population (*N*=1197, 84.4%).

### Randomisation of MMR-proficient CRCs

The 1197 MMR-proficient CRCs were randomly assigned into two groups, Study Group 2A (*N*=599) and Study Group 2B (*N*=598). Study Group 2A was used to determine the most relevant cutoff scores above which a tumour should be considered to overexpress EGFR for each clinicopathological feature. The associations of EGFR expression at the proposed cutoff scores with T stage, N stage, tumour grade, vascular invasion and survival were investigated on Study Group 2B.

### Statistical analysis

#### Inter-observer reliability of the scoring method

The reproducibility of the semiquantitative scoring method in both rectal tumour biopsies and TMA CRC punches was assessed among three pathologists and analysed using the intraclass correlation coefficient (ICC) (Shrout and Fleiss, 1979; Zlobec *et al*, 2006a). The ICC is defined as the ratio of the between-subject variance over the between-subject+within subject variances and has previously been used to assess agreement of IHC scores (Kirkegaard *et al*, 2006).

#### Selecting the cutoff scores for EGFR ‘positivity’

The selection of cutoff scores for EGFR expression in both Study group 1 and 2A were based on ROC curve analysis (Zlobec *et al*, 2006b). At each score, the sensitivity and specificity for the outcome being studied were plotted thus generating a ROC curve. The score located closest to the point with both maximum sensitivity and specificity, that is, the point (0.0, 1.0) on the curve, was selected as the cutoff score leading to the greatest number of tumours which were correctly classified as having or not having the outcome. To use ROC curve analysis, the clinical and tumour characteristics must be binary and were therefore dichotomized. For Study group 1 two analyses were performed, the first to predict complete pathological response (complete response *vs* partial or no response) and any response (complete or partial response *vs* no response). For Study group 2, T stage was dichotomized as early (T1+T2) or late (T3+T4), N stage as N0 (no lymph node involvement) or >N0 (any lymph node involvement), tumour grade as low (G1+G2) or high (G3), vascular invasion as absent or present and survival as death due to CRC or other (censored, alive or death from other causes).

#### Reproducibility of ROC curve analysis

To determine whether ROC curve analysis was a reproducible method for selecting the cutoff scores for EGFR, ROC curves were generated for each independent pathologist and clinicopathological feature. In addition, 100-bootstrapped replications were performed to resample the data and determine the reliability of the cutoff scores obtained by each scorer. With bootstrapping, 100 resamples of equal size are created and ROC curve analysis is performed for each subgroup. Finally, the cutoff scores from each pathologist were averaged and subsequently used to determine the association of EGFR overexpression and the clinicopathological features on Study Group 2B. The most frequently obtained cutoff score over the 100 resamples the area under the ROC curve (AUC) and 95% CI were acquired for each analysis. Area under the ROC curves summarise the discriminatory power of EGFR for the outcome with values of 0.5 indicating low power and those closer to 1.0 higher power.

#### Association with clinicopathological features at the respective cutoffs

To determine the association of EGFR expression and tumour response, logistic regression analysis was performed. The odds ratio (OR) and 95% CI were obtained. The *χ*^2^ test was used to evaluate EGFR expression with T stage, N stage, tumour grade and vascular invasion. Survival analysis was carried out using the Kaplan–Meier method and log-rank test. Cox proportional hazards regression was used in multivariate survival analysis to identify the prognostic value of EGFR independently of T stage, N stage, tumour grade, vascular invasion and age. All analyses were carried out using SAS (The SAS Institute, Cary, NC, USA). Receiver operating characteristic curves were plotted using SPSS.

## RESULTS

### Tumour characteristics

#### Study Group 1

**Figure 1:**
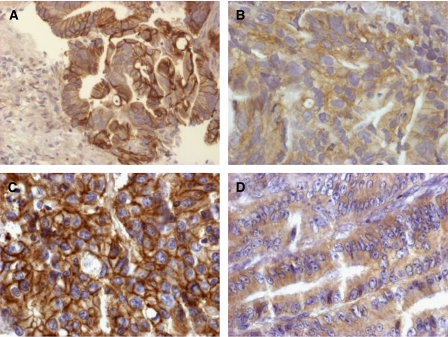
Predominantly membranous (**A**) and cytoplasmic (**B**) EGFR expression in rectal adenocarcinoma (× 40). Membranous (**C**) and cytoplasmic (**D**) EGFR staining in TMA punches of moderately differentiated MMR-proficient CRCs (× 40).

Twenty-seven tumours (32.9%) were completely responsive to preoperative HDREB whereas 29 (35.4%) were non-responsive and 30 (36.6%) were partially responsive to therapy. Fifteen tumours (18.3%) showed no immunoreactivity (0% staining) for EGFR whereas 67 (81.7%) displayed either membranous or cytoplasmic positivity (Figure 1A and B). More than 90% of patients were staged as cT3.

#### Study Group 2

EGFR immunoreactivity was evaluated in 1032 MMR-proficient CRCs. One hundred and sixty-five cases were not assessed owing to the absence of tissue or tumour. Absence of staining was found in 367 (35.6%) cases whereas membranous and/or cytoplasmic staining was described in 64.4% (Figure 1C and D).

#### Inter-observer agreement

The ICCs obtained by analysing the rectal tumour biopsies and TMA CRC punches were 0.71 and 0.86, respectively.

#### ROC curve analysis

**Figure 2:**
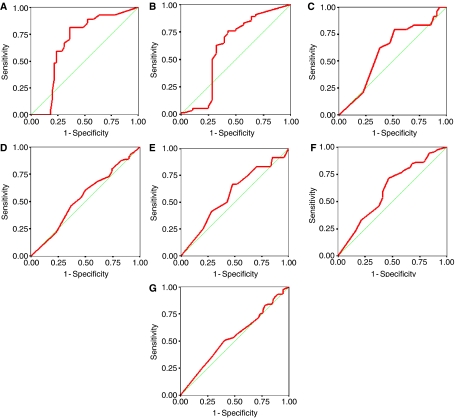
ROC curves plotted for each clinicopathological feature using scores from one of the three pathologists (**A**) complete tumour response, (**B**) complete or partial tumour response, (**C**) T stage, (**D**) N stage, (**E**) tumour grade, (**F**) vascular invasion and (**G**) 10-year survival. Arrows indicate the closest point on the ROC curves to the point (0.0, 1.0), which correspond to the selected cut-off score.

The cutoff values for EGFR positivity or overexpression were determined by ROC curve analysis. For the three pathologists, a cutoff score was obtained (Table 1) for each clinicopathological feature. Values were highly consistent between pathologists. The average EGFR cutoff scores were obtained and included 18% for predicting complete response, 15% for predicting complete or partial response, 85% for T stage, and 10-year survival, 75% for N stage, 82% for tumour grade and 80% for vascular invasion. The ROC curves for each outcome are shown in Figure 2.

#### Association of EGFR and clinicopathological features (Table 2)

Positive EGFR expression (>18% tumour cell staining) was significantly associated with complete pathological response to preoperative HDREB (*P*-value <0.001; OR (95% CI)=7.12 (2.3–21.7)). Complete or partial tumour response was more frequently associated with positive EGFR expression (>15% tumour cell staining) (*P*-value=0.008; OR (95% CI)=3.59 (1.37–9.43)).

Overexpression of EGFR (>85% tumour cell staining) was more frequently found in tumours with late T stage, although this difference was only marginally significant (*P*=0.069). No association between EGFR overexpression and N stage (*P*-value=0.792) or vascular invasion (*P*=0.753) was found. A marginally significant difference in tumour grade with EGFR overexpression was observed (*P*=0.051).

**Figure 3:**
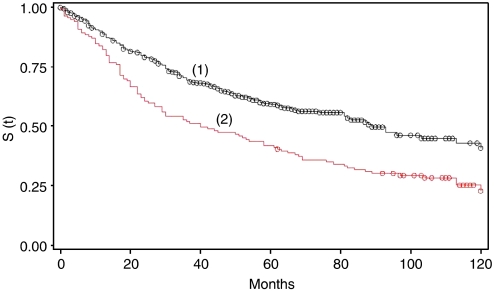
Kaplan–Meier survival curve for MMR-proficient CRCs with (1) <85% EGFR staining, (2) ⩾85% EGFR staining.

Tumours with <85% EGFR staining had a significantly better survival time (87.0 months (69.0–103.0)) (*P*<0.001) compared to tumours overexpressing the protein (35.0 months (23.0–58.0)) (Figure 3). In a multivariate survival analysis adjusting for T stage, N stage, tumour grade, vascular invasion and age, EGFR overexpression was independently associated with worse survival time (*P*<0.001) (HR (95% CI)=1.93 (1.44–2.57)).

## DISCUSSION

The predictive and prognostic value of EGFR in CRC varies significantly in the literature. Several reasons have been suggested for this discrepancy such as non-comparable study populations (Spano *et al*, 2005a), variability in protocols, fixation and antibodies (Atkins *et al*, 2004) and the lack of a uniform scoring system (Penault-Llorca *et al*, 2005; Italiano, 2006; Walker, 2006).

The aim of this study was to determine the predictive and prognostic value of EGFR in CRC based on cutoff scores selected to maximise the clinical utility of EGFR findings by IHC. EGFR expression and tumour response to a novel preoperative radiation protocol, namely HDREB, was evaluated on whole tumour biopsy specimens. In addition, 1197 CRCs from TMA punches were randomised into two subgroups, the first used to select the cutoff scores for EGFR overexpression, the second to analyse EGFR overexpression and its association with tumour progression and survival. The TMA approach is an accepted tool of investigation, in particular with large sample sizes (Moch *et al*, 1999; Barlund *et al*, 2000; Nocito *et al*, 2001; Simon *et al*, 2001; Torhorst *et al*, 2001; Sauter *et al*, 2003; Goethals *et al*, 2006).

The evaluation of immunoreactivity was carried out semiquantitatively by scoring the percentage of positive tumour cells in both rectal tumour biopsy specimens and TMA punches. We have previously shown that this scoring method leads to a more complete assessment of the prognostic value of several tumour markers in CRC when compared to an evaluation system based on arbitrarily determined ‘positive’ or ‘negative’ scores (Lugli *et al*, 2006a, 2006b, 2006c, 2006d). We have also shown that this scoring method is reproducible among pathologists in rectal cancer using the ICC which has recently been proposed as a method for determining inter-observer variation of semicontinuous immunohistochemical scores (Kirkegaard *et al*, 2006; Zlobec *et al*, 2006a). An ICC greater than 0.7 should be regarded as the acceptable minimum standard for declaring reliability (Kirkegaard *et al*, 2006). In this study, we again validate this scoring method for EGFR among three independent pathologists in rectal cancer biopsies (ICC=0.71) and TMA punches of CRC (ICC=0.86).

ROC curves are commonly used in clinical oncology to determine the threshold value above which a test result should be considered positive for some outcome (Hanley, 1989; Al-Homoud *et al*, 2004; Carpelan-Holmstrom *et al*, 2004; Chen *et al*, 2005; Reid *et al*, 2005; Lind *et al*, 2006; Linke *et al*, 2006; Punglia *et al*, 2006). We applied the same principle in this study to determine the cutoff scores above which EGFR should be considered overexpressed (Zlobec *et al*, 2006b). The reproducibility of this method was validated by generating ROC curves for each of the three pathologist's scores in addition to resampling of the data. The results of this study demonstrate that the selected cutoff scores for each clinicopathological feature were highly consistent among pathologists.

To obtain the best estimate of the EGFR expression in each tumour, the three cutoff scores from each pathologist were averaged. The cutoff score varied with the end point under investigation. EGFR was considered to be overexpressed when more than 15% of cells were stained when evaluating rectal tumour response to HDREB but was significantly greater when analysing features related to tumour progression and survival (⩾75% staining). This difference in cutoff scores may be due to the selection of patients into each Study Group. The rectal cancer patients in Study Group 1 had predominantly cT3 tumours whereas those in Study Group 2 were unselected and included tumours of all T stages. The distribution of EGFR scores in both study groups varied considerably with those in Study group 1 ranging from 0 to 90% with only 5% of tumours expressing EGFR in more than 80% of tumour cells.

The findings of this study underline the fact that the selection of cutoff scores for positivity should be performed for the specific end point under investigation. The cutoff score of 15% is therefore specific for predicting complete response in patients undergoing treatment with preoperative HDREB and may not be generalisable to other forms of radiotherapy for which cutoff scores can be established.

When investigating outcomes, such as response to anti-EGFR therapy, it may be more beneficial to choose a cutoff score leading to high sensitivity rather than specificity for tumour response to select the greatest number of potentially responsive candidates for treatment. In this study, the cutoff score was selected such that it maximised the number of correctly classified tumours with and without the end point being under evaluation (maximum sensitivity with minimal loss of specificity).

At the selected cutoff scores, EGFR overexpression was significantly associated with improved response to preoperative HDREB. Complete pathological response was more than seven times more likely to occur in tumours overexpressing EGFR whereas complete or partial response was found to occur nearly four times more often in these cases. These results are in line with reports in head and neck squamous cell carcinoma investigating the predictive value of EGFR using a high-dose rate approach (Eriksen *et al*, 2004; Bentzen *et al*, 2005).

EGFR overexpression in MMR-proficient CRC was not associated with N stage or vascular invasion and led to marginally significant associations with T stage and tumour grade. These results are supported by similar findings by other groups that have shown no relationship between EGFR overexpression and disease evolution (Yasui *et al*, 1988; Moorghen *et al*, 1990; Koenders *et al*, 1992; Saeki *et al*, 1995; Goldstein and Armin, 2001; Yarden and Sliwkowski, 2001; McKay *et al*, 2002; Spano *et al*, 2005b). However, patients with EGFR overexpressing tumours (⩾85% tumour cell staining) demonstrated a significantly worse prognosis (35.0 months (23.0–58.0)) than those with no overexpression (87.0 months (69.0–103.0)). Previous reports also support these findings (Gross *et al*, 1991; Mayer *et al*, 1993; Khorana *et al*, 2003; Resnick *et al*, 2004; Galizia *et al*, 2006). Moreover, EGFR in this study was found to predict worse survival in a multivariate analysis independently of known adverse prognostic factors including T stage, N stage and vascular invasion. These results indicate that EGFR expression evaluated at a cutoff of 85% could be used as a prognostic marker in addition to pathological staging.

In conclusion, EGFR is a predictive marker of response to preoperative HDREB in rectal cancers and an independent adverse prognostic factor in MMR-proficient CRC. The combination of semiquantitative evaluation of EGFR expression and ROC curve analysis which was validated in this study proves to be a reproducible method for selecting the cutoff scores for EGFR overexpression in CRC.

## Figures and Tables

**Table 1:** Most frequently obtained cutoff score (mode), and area under the ROC curve (AUC (95% CI)) for each pathologist and clinicopathological feature

		**Pathologist**			**Average**
	**ROC features**	**No. 1**	**No. 2**	**No. 3**	**cutoff scores**
Predicting CR	Cutoff score	10%	25%	20%	18%
	AUC (95% CI)	0.63 (0.51–0.75)	0.654 (0.53–0.78)	0.70 (0.59–0.82)	
Predicting CR or PR	Cutoff score	10%	15%	20%	15%
	AUC (95% CI)	0.571 (0.43–0.71)	0.652 (0.51–0.79)	0.60 (0.46–0.75)	
T stage	Cutoff score	90%	85%	80%	85%
	AUC (95% CI)	0.594 (0.48–0.71)	0.579 (0.45–0.70)	0.533 (0.47–0.60)	
N stage	Cutoff score	80%	75%	70%	75%
	AUC (95% CI)	0.536 (0.45–0.62)	0.552 (0.47–0.64)	0.505 (0.45–0.55)	
Tumour grade	Cut-off score	90%	85%	60%	82%
	AUC (95% CI)	0.587 (0.41–0.77)	0.574 (0.41–0.74)	0.513 (0.43–0.60)	
Vascular invasion	Cutoff score	90%	75%	80%	80%
	AUC (95% CI)	0.548 (0.45–0.64)	0.61 (0.52–0.70)	0.515 (0.46–0.57)	
10-year survival	Cutoff score	85%	90%	80%	85%
	AUC (95% CI)	0.523 (0.44–0.61)	0.536 (0.45–0.62)	0.501 (0.44–0.56)	

AUC=area under ROC curve; CR=complete response, PR=partial response; ROC=receiver operating characteristic.

**Table 2:** Association of EGFR expression and clinicopathological features

	**Cutoff**	**Below cutoff *N* (%)**	**Above cutoff *N* (%)**	***P*-value**
*Predicting CR*				
CR	18%	6 (14.6)	21 (51.2)	<0.001
PR or NR		35 (85.4)	20 (48.8)	
				
*Predicting CR or PR*				
CR or PR	15%	20 (51.3)	34 (79.1)	0.008
NR		19 (48.7)	9 (20.9)	
				
*T stage*				
Early (T1+T2)	85%	118 (24.1)	14 (15.4)	0.069
Late (T3+T4)		372 (75.9)	77 (84.6)	
				
*N stage*				
N0	75%	244 (52.4)	54 (50.9)	0.792
>N0		222 (47.6)	52 (49.1)	
				
*Tumour grade*				
G1+G2	82%	431 (87.8)	89 (94.7)	0.051
G3		60 (12.2)	5 (5.3)	
				
*Vascular invasion*				
Presence	80%	133 (27.6)	30 (29.1)	0.753
Absence		349 (72.4)	73 (70.9)	
				
*10-year survival*				
Median (95% CI) (months)	85%	87.0 (69.0–103.0)	35.0 (23.0–58.0)	<0.001

CR=complete response, PR=partial response.

Cutoff scores were obtained by ROC curve analysis performed on the average EGFR scores.

## References

[bib1] Al-Homoud S, Purkayastha S, Aziz O, Smith JJ, Thompson MD, Darzi AW, Stamatakis JD, Tekkis PP (2004) Evaluating operative risk in colorectal cancer surgery: ASA and POSSUM-based predictive models. Surg Oncol 13: 83–92 15572090 10.1016/j.suronc.2004.08.006

[bib2] Atkins D, Reiffen KA, Tegtmeier CL, Winther H, Bonato MS, Storkel S (2004) Immunohistochemical detection of EGFR in paraffin-embedded tumour tissues: variation in staining intensity due to choice of fixative and storage time of tissue sections. J Histochem Cytochem 52: 893–901 15208356 10.1369/jhc.3A6195.2004

[bib3] Azria D, Bibeau F, Barbier N, Zouhair A, Lemanski C, Rouanet P, Ychou M, Senesse P, Ozsahin M, Pelegrin A, Dubois JB, Thezenas S (2005) Prognostic impact of epidermal growth factor receptor (EGFR) expression on loco-regional recurrence after preoperative radiotherapy in rectal cancer. BMC Cancer 5: 62 15967033 10.1186/1471-2407-5-62 PMC1185521

[bib4] Barlund M, Forozan F, Kononen J, Bubendorf L, Chen Y, Bittner ML, Torhorst J, Haas P, Bucher C, Sauter G, Kallioniemi OP, Kallioniemi A (2000) Detecting activation of ribosomal protein S6 kinase by complementary DNA and tissue microarray analysis. J Natl Cancer Inst 92: 1252–1259 10922410 10.1093/jnci/92.15.1252

[bib5] Baselga J. (2002) Targeting the epidermal growth factor receptor with tyrosine kinase inhibitors: small molecules, big hopes. J Clin Oncol 20: 2217–2219 11980990 10.1200/JCO.2002.20.9.2217

[bib6] Bentzen SM, Atasoy BM, Daley FM, Dische S, Richman PI, Saunders MI, Trott KR, Wilson GD (2005) Epidermal growth factor receptor expression in pretreatment biopsies from head and neck squamous cell carcinoma as a predictive factor for a benefit from accelerated radiation therapy in a randomized controlled trial. J Clin Oncol 23: 5560–5567 16110017 10.1200/JCO.2005.06.411

[bib7] Bibeau F, Boissiere-Michot F, Sabourin JC, Gourgou-Bourgade S, Radal M, Penault-Llorca F, Rochaix P, Arnould L, Bralet MP, Azria D, Ychou M (2006) Assessment of epidermal growth factor receptor (EGFR) expression in primary colorectal carcinomas and their related metastases on tissue sections and tissue microarray. Virchows Arch 449: 281–287 16865406 10.1007/s00428-006-0247-9 PMC1888717

[bib8] Carpelan-Holmstrom M, Louhimo J, Stenman UH, Alfthan H, Jarvinen H, Haglund C (2004) CEA, CA 242, CA 19-9, CA 72-4 and hCGbeta in the diagnosis of recurrent colorectal cancer. Tumour Biol 25: 228–234 15627885 10.1159/000081385

[bib9] Chen Y, Hyrien O, Williams J, Okunieff P, Smudzin T, Rubin P (2005) Interleukin (IL)-1A and IL-6: applications to the predictive diagnostic testing of radiation pneumonitis. Int J Radiat Oncol Biol Phys 62: 260–266 15850931 10.1016/j.ijrobp.2005.01.041

[bib10] Chua DT, Sham JS, Kwong DL, Choy DT, Au GK, Wu PM (1996) Prognostic value of paranasopharyngeal extension of nasopharyngeal carcinoma. A significant factor in local control and distant metastasis. Cancer 78: 202–210 8673993 10.1002/(SICI)1097-0142(19960715)78:2<202::AID-CNCR3>3.0.CO;2-N

[bib11] Cunningham D, Humblet Y, Siena S, Khayat D, Bleiberg H, Santoro A, Bets D, Mueser M, Harstrick A, Verslype C, Chau I, Van Cutsem E (2004) Cetuximab monotherapy and cetuximab plus irinotecan in irinotecan-refractory metastatic colorectal cancer. N Engl J Med 351: 337–345 15269313 10.1056/NEJMoa033025

[bib12] Eriksen JG, Steiniche T, Askaa J, Alsner J, Overgaard J (2004) The prognostic value of epidermal growth factor receptor is related to tumour differentiation and the overall treatment time of radiotherapy in squamous cell carcinomas of the head and neck. Int J Radiat Oncol Biol Phys 58: 561–566 14751528 10.1016/j.ijrobp.2003.09.043

[bib13] Galizia G, Lieto E, Ferraraccio F, De Vita F, Castellano P, Orditura M, Imperatore V, La Mura A, La Manna G, Pinto M, Catalano G, Pignatelli C, Ciardiello F (2006) Prognostic significance of epidermal growth factor receptor expression in colon cancer patients undergoing curative surgery. Ann Surg Oncol 13: 823–835 16614884 10.1245/ASO.2006.05.052

[bib14] Gamboa-Dominguez A, Dominguez-Fonseca C, Quintanilla-Martinez L, Reyes-Gutierrez E, Green D, Angeles-Angeles A, Busch R, Hermannstadter C, Nahrig J, Becker KF, Becker I, Hofler H, Fend F, Luber B (2004) Epidermal growth factor receptor expression correlates with poor survival in gastric adenocarcinoma from Mexican patients: a multivariate analysis using a standardized immunohistochemical detection system. Mod Pathol 17: 579–587 15073595 10.1038/modpathol.3800085

[bib15] Goethals L, Debucquoy A, Perneel C, Geboes K, Ectors N, De Schutter H, Penninckx F, McBride WH, Begg AC, Haustermans KM (2006) Hypoxia in human colorectal adenocarcinoma: comparison between extrinsic and potential intrinsic hypoxia markers. Int J Radiat Oncol Biol Phys 65: 246–254 16618579 10.1016/j.ijrobp.2006.01.007

[bib16] Goldstein NS, Armin M (2001) Epidermal growth factor receptor immunohistochemical reactivity in patients with American Joint Committee on Cancer Stage IV colon adenocarcinoma: implications for a standardized scoring system. Cancer 92: 1331–1346 11571750 10.1002/1097-0142(20010901)92:5<1331::aid-cncr1455>3.0.co;2-m

[bib17] Grant S, Qiao L, Dent P (2002) Roles of ERBB family receptor tyrosine kinases, and downstream signaling pathways, in the control of cell growth and survival. Front Biosci 7: d376–d389 11815285 10.2741/grant

[bib18] Gross ME, Zorbas MA, Danels YJ, Garcia R, Gallick GE, Olive M, Brattain MG, Boman BM, Yeoman LC (1991) Cellular growth response to epidermal growth factor in colon carcinoma cells with an amplified epidermal growth factor receptor derived from a familial adenomatous polyposis patient. Cancer Res 51: 1452–1459 1847663

[bib19] Hampel H, Stephens JA, Pukkala E, Sankila R, Aaltonen LA, Mecklin JP, de la Chapelle A (2005) Cancer risk in hereditary nonpolyposis colorectal cancer syndrome: later age of onset. Gastroenterology 129: 415–421 16083698 10.1016/j.gastro.2005.05.011

[bib20] Hanley J (1989) Receiver operating characteristic (ROC) methodology: the state of the art. Critical Rev Diagn Imagin 29: 307–337 2667567

[bib21] Herbst RS (2004) Review of epidermal growth factor receptor biology. Int J Radiat Oncol Biol Phys 59: 21–26 10.1016/j.ijrobp.2003.11.041 15142631

[bib22] Italiano A (2006) Targeting the epidermal growth factor receptor in colorectal cancer: advances and controversies. Oncology 70: 161–167 16675911 10.1159/000093092

[bib23] Italiano A, Saint-Paul MC, Caroli-Bosc FX, Francois E, Bourgeon A, Benchimol D, Gugenheim J, Michiels JF (2005) Epidermal growth factor receptor (EGFR) status in primary colorectal tumours correlates with EGFR expression in related metastatic sites: biological and clinical implications. Ann Oncol 16: 1503–1507 15980160 10.1093/annonc/mdi282

[bib24] Italiano A, Vandenbos FB, Otto J, Mouroux J, Fontaine D, Marcy PY, Cardot N, Thyss A, Pedeutour F (2006) Comparison of the epidermal growth factor receptor gene and protein in primary non-small-cell-lung cancer and metastatic sites: implications for treatment with EGFR-inhibitors. Ann Oncol 17: 981–985 16524970 10.1093/annonc/mdl038

[bib25] Jonjic N, Kovac K, Krasevic M, Valkovic T, Ernjak N, Sasso F, Melato M (1997) Epidermal growth factor-receptor expression correlates with tumour cell proliferation and prognosis in gastric cancer. Anticancer Res 17: 3883–3888 9427797

[bib26] Kainulainen V, Sundvall M, Maatta JA, Santiestevan E, Klagsbrun M, Elenius K (2000) A natural ErbB4 isoform that does not activate phosphoinositide 3-kinase mediates proliferation but not survival or chemotaxis. J Biol Chem 275: 8641–8649 10722704 10.1074/jbc.275.12.8641

[bib27] Khorana AA, Ryan CK, Cox C, Eberly S, Sahasrabudhe DM (2003) Vascular endothelial growth factor, CD68, and epidermal growth factor receptor expression and survival in patients with stage II and stage III colon carcinoma: a role for the host response in prognosis. Cancer 97: 960–968 12569594 10.1002/cncr.11152

[bib28] Kirkegaard T, Edwards J, Tovey S, McGlynn LM, Krishna SN, Mukherjee R, Tam L, Munro AF, Dunne B, Bartlett JM (2006) Observer variation in immunohistochemical analysis of protein expression, time for a change? Histopathology 48: 787–794 16722926 10.1111/j.1365-2559.2006.02412.x

[bib29] Koenders PG, Peters WH, Wobbes T, Beex LV, Nagengast FM, Benraad TJ (1992) Epidermal growth factor receptor levels are lower in carcinomatous than in normal colorectal tissue. Br J Cancer 65: 189–192 1739615 10.1038/bjc.1992.39 PMC1977742

[bib30] Lind PA, Wennberg B, Gagliardi G, Rosfors S, Blom-Goldman U, Lidestahl A, Svane G (2006) ROC curves and evaluation of radiation-induced pulmonary toxicity in breast cancer. Int J Radiat Oncol Biol Phys 64: 765–770 16257129 10.1016/j.ijrobp.2005.08.011

[bib31] Linke SP, Bremer TM, Herold CD, Sauter G, Diamond C (2006) A multimarker model to predict outcome in tamoxifen-treated breast cancer patients. Clin Cancer Res 12: 1175–1183 16489071 10.1158/1078-0432.CCR-05-1562

[bib32] Lugli A, Zlobec I, Baker K, Minoo P, Tornillo L, Terracciano L, Jass J (2006a) Prognostic significance of mucins in colorectal cancer with different DNA mismatch-repair status. J Clin Pathol 30 June [E-pub ahead of print] 10.1136/jcp.2006.039552 PMC1994556 16816167

[bib33] Lugli A, Zlobec I, Gunthert U, Minoo P, Baker K, Tornillo L, Terracciano L, Jass JR (2006b) Overexpression of the receptor for hyaluronic acid mediated motility is an independent adverse prognostic factor in colorectal cancer. Mod Pathol 19: 1302–1309 16763611 10.1038/modpathol.3800648

[bib34] Lugli A, Zlobec I, Minoo P, Baker K, Tornillo L, Terracciano L, Jass J (2006c) Prognostic significance of the wnt signaling pathway molecules APC, b-catenin and E-cadherin in colorectal cancer. Histopathology In press 10.1111/j.1365-2559.2007.02620.x 17448021

[bib35] Lugli A, Zlobec I, Minoo P, Baker K, Tornillo L, Terracciano L, Jass JR (2006d) Role of the mitogen-activated protein kinase and phosphoinositide 3-kinase/AKT pathways downstream molecules, phosphorylated extracellular signal-regulated kinase, and phosphorylated AKT in colorectal cancer-A tissue microarray-based approach. Hum Pathol 37: 1022–1031 16867865 10.1016/j.humpath.2006.03.002

[bib36] Mayer A, Takimoto M, Fritz E, Schellander G, Kofler K, Ludwig H (1993) The prognostic significance of proliferating cell nuclear antigen, epidermal growth factor receptor, and mdr gene expression in colorectal cancer. Cancer 71: 2454–2460 8095852 10.1002/1097-0142(19930415)71:8<2454::aid-cncr2820710805>3.0.co;2-2

[bib37] McKay JA, Murray LJ, Curran S, Ross VG, Clark C, Murray GI, Cassidy J, McLeod HL (2002) Evaluation of the epidermal growth factor receptor (EGFR) in colorectal tumours and lymph node metastases. Eur J Cancer 38: 2258–2264 12441262 10.1016/s0959-8049(02)00234-4

[bib38] Moch H, Schraml P, Bubendorf L, Mirlacher M, Kononen J, Gasser T, Mihatsch MJ, Kallioniemi OP, Sauter G (1999) High-throughput tissue microarray analysis to evaluate genes uncovered by cDNA microarray screening in renal cell carcinoma. Am J Pathol 154: 981–986 10233835 10.1016/S0002-9440(10)65349-7 PMC1866554

[bib39] Moorghen M, Ince P, Finney KJ, Watson AJ, Harris AL (1990) Epidermal growth factor receptors in colorectal carcinoma. Anticancer Res 10: 605–611 2195985

[bib40] Neal DE, Sharples L, Smith K, Fennelly J, Hall RR, Harris AL (1990) The epidermal growth factor receptor and the prognosis of bladder cancer. Cancer 65: 1619–1625 2311071 10.1002/1097-0142(19900401)65:7<1619::aid-cncr2820650728>3.0.co;2-q

[bib41] Nicholson S, Richard J, Sainsbury C, Halcrow P, Kelly P, Angus B, Wright C, Henry J, Farndon JR, Harris AL (1991) Epidermal growth factor receptor (EGFr); results of a 6 year follow-up study in operable breast cancer with emphasis on the node negative subgroup. Br J Cancer 63: 146–150 1846551 10.1038/bjc.1991.30 PMC1971641

[bib42] Nocito A, Bubendorf L, Tinner EM, Suess K, Wagner U, Forster T, Kononen J, Fijan A, Bruderer J, Schmid U, Ackermann D, Maurer R, Alund G, Knonagel H, Rist M, Anabitarte M, Hering F, Hardmeier T, Schoenenberger AJ, Flury R, Jager P, Fehr JL, Schraml P, Moch H, Mihatsch MJ, Gasser T, Sauter G (2001) Microarrays of bladder cancer tissue are highly representative of proliferation index and histological grade. J Pathol 194: 349–357 11439368 10.1002/1096-9896(200107)194:3<349::AID-PATH887>3.0.CO;2-D

[bib43] Penault-Llorca F, Bibeau F, Arnould L, Bralet MP, Rochaix P, Sabourin JC (2005) EGFR expression in colorectal cancer and role in tumourigenesis. Bull Cancer 92: S5–11 16387663

[bib44] Prewett MC, Hooper AT, Bassi R, Ellis LM, Waksal HW, Hicklin DJ (2002) Enhanced antitumour activity of anti-epidermal growth factor receptor monoclonal antibody IMC-C225 in combination with irinotecan (CPT-11) against human colorectal tumour xenografts. Clin Cancer Res 8: 994–1003 12006511

[bib45] Punglia RS, D'Amico AV, Catalona WJ, Roehl KA, Kuntz KM (2006) Impact of age, benign prostatic hyperplasia, and cancer on prostate-specific antigen level. Cancer 106: 1507–1513 16518812 10.1002/cncr.21766

[bib46] Radinsky R (1995) Modulation of tumour cell gene expression and phenotype by the organ-specific metastatic environment. Cancer Metastasis Rev 14: 323–338 8821093 10.1007/BF00690601

[bib47] Radinsky R, Risin S, Fan D, Dong Z, Bielenberg D, Bucana CD, Fidler IJ (1995) Level and function of epidermal growth factor receptor predict the metastatic potential of human colon carcinoma cells. Clin Cancer Res 1: 19–31 9815883

[bib48] Reid JF, Lusa L, De Cecco L, Coradini D, Veneroni S, Daidone MG, Gariboldi M, Pierotti MA (2005) Limits of predictive models using microarray data for breast cancer clinical treatment outcome. J Natl Cancer Inst 97: 927–930 15956654 10.1093/jnci/dji153

[bib49] Resnick MB, Routhier J, Konkin T, Sabo E, Pricolo VE (2004) Epidermal growth factor receptor, c-MET, beta-catenin, and p53 expression as prognostic indicators in stage II colon cancer: a tissue microarray study. Clin Cancer Res 10: 3069–3075 15131045 10.1158/1078-0432.ccr-03-0462

[bib50] Saeki T, Salomon DS, Johnson GR, Gullick WJ, Mandai K, Yamagami K, Moriwaki S, Tanada M, Takashima S, Tahara E (1995) Association of epidermal growth factor-related peptides and type I receptor tyrosine kinase receptors with prognosis of human colorectal carcinomas. Jpn J Clin Oncol 25: 240–249 8523820

[bib51] Sauter G, Simon R, Hillan K (2003) Tissue microarrays in drug discovery. Nat Rev Drug Discov 2: 962–972 14654795 10.1038/nrd1254

[bib52] Shrout PE, Fleiss JL (1979) Intra-class correlations: uses in assessing rater reliability. Psychol Bull 2: 420–428 10.1037//0033-2909.86.2.420 18839484

[bib53] Simon R, Nocito A, Hubscher T, Bucher C, Torhorst J, Schraml P, Bubendorf L, Mihatsch MM, Moch H, Wilber K, Schotzau A, Kononen J, Sauter G (2001) Patterns of her-2/neu amplification and overexpression in primary and metastatic breast cancer. J Natl Cancer Inst 93: 1141–1146 11481385 10.1093/jnci/93.15.1141

[bib54] Spano JP, Fagard R, Soria JC, Rixe O, Khayat D, Milano G (2005a) Epidermal growth factor receptor signaling in colorectal cancer: preclinical data and therapeutic perspectives. Ann Oncol 16: 189–194 15668269 10.1093/annonc/mdi057

[bib55] Spano JP, Lagorce C, Atlan D, Milano G, Domont J, Benamouzig R, Attar A, Benichou J, Martin A, Morere JF, Raphael M, Penault-Llorca F, Breau JL, Fagard R, Khayat D, Wind P (2005b) Impact of EGFR expression on colorectal cancer patient prognosis and survival. Ann Oncol 16: 102–108 15598946 10.1093/annonc/mdi006

[bib56] Tateishi M, Ishida T, Mitsudomi T, Kaneko S, Sugimachi K (1990) Immunohistochemical evidence of autocrine growth factors in adenocarcinoma of the human lung. Cancer Res 50: 7077–7080 2208175

[bib57] Thant AA, Nawa A, Kikkawa F, Ichigotani Y, Zhang Y, Sein TT, Amin AR, Hamaguchi M (2000) Fibronectin activates matrix metalloproteinase-9 secretion via the MEK1-MAPK and the PI3K-Akt pathways in ovarian cancer cells. Clin Exp Metastasis 18: 423–428 11467775 10.1023/a:1010921730952

[bib58] Torhorst J, Bucher C, Kononen J, Haas P, Zuber M, Kochli OR, Mross F, Dieterich H, Moch H, Mihatsch M, Kallioniemi OP, Sauter G (2001) Tissue microarrays for rapid linking of molecular changes to clinical endpoints. Am J Pathol 159: 2249–2256 11733374 10.1016/S0002-9440(10)63075-1 PMC1850582

[bib59] Umemura S, Itoh J, Itoh H, Serizawa A, Saito Y, Suzuki Y, Tokuda Y, Tajima T, Osamura RY (2004) Immunohistochemical evaluation of hormone receptors in breast cancer: which scoring system is suitable for highly sensitive procedures? Appl Immunohistochem Mol Morphol 12: 8–13 15163012 10.1097/00129039-200403000-00002

[bib60] Vuong T, Belliveau PJ, Michel RP, Moftah BA, Parent J, Trudel JL, Reinhold C, Souhami L (2002) Conformal preoperative endorectal brachytherapy treatment for locally advanced rectal cancer: early results of a phase I/II study. Dis Colon Rectum 45: 1486–1493, discussion 1493–1495 12432296 10.1007/s10350-004-6455-y

[bib61] Vuong T, Devic S, Moftah B, Evans M, Podgorsak EB (2005) High-dose-rate endorectal brachytherapy in the treatment of locally advanced rectal carcinoma: technical aspects. Brachytherapy 4: 230–235 16182224 10.1016/j.brachy.2005.03.006

[bib62] Walker RA (2006) Quantification of immunohistochemistry-issues concerning methods, utility and semiquantitative assessment I. Histopathology 49: 406–410 16978204 10.1111/j.1365-2559.2006.02514.x

[bib63] Wan CW, McKnight MK, Brattain DE, Brattain MG, Yeoman LC (1988) Different epidermal growth factor growth responses and receptor levels in human colon carcinoma cell lines. Cancer Lett 43: 139–143 3264518 10.1016/0304-3835(88)90226-1

[bib64] Yarden Y, Sliwkowski MX (2001) Untangling the ErbB signalling network. Nat Rev Mol Cell Biol 2: 127–137 11252954 10.1038/35052073

[bib65] Yasui W, Sumiyoshi H, Hata J, Kameda T, Ochiai A, Ito H, Tahara E (1988) Expression of epidermal growth factor receptor in human gastric and colonic carcinomas. Cancer Res 48: 137–141 2446740

[bib66] Zlobec I, Steele R, Michel RP, Compton CC, Lugli A, Jass JR (2006a) Scoring of p53, VEGF, Bcl-2 and APAF-1 immunohistochemistry and interobserver reliability in colorectal cancer. Mod Pathol 19: 1236–1242 16741523 10.1038/modpathol.3800642

[bib67] Zlobec I, Steele R, Terracciano L, Jass J, Lugli A (2006b) Selecting immunohistochemical cut-off scores for novel biomarkers of progression and survival in colorectal cancer. J Clin Pathol, 20 December [E-pub ahead of print] 10.1136/jcp.2006.044537 PMC2014838 17182662

